# Management of pediatric vanishing testes syndrome based on pathological diagnosis: a single-center retrospective study

**DOI:** 10.1038/s41598-024-59583-6

**Published:** 2024-04-24

**Authors:** Chang-Kun Mao, Yuan-Fang, Yong-Sheng Cao

**Affiliations:** 1 Department of Urology, Anhui Provincial Children’s Hospital, No. 39 East Wangjiang Road, Hefei, 230022 Anhui China; 2 Department of Pathology, Anhui Provincial Children’s Hospital, No. 39 East Wangjiang Road, Hefei, 230022 Anhui China

**Keywords:** Medical research, Urology

## Abstract

This study aims to explore the optimal management strategy for pediatric vanishing testes syndrome (VTS) based on pathological characteristics. We retrospectively analyzed clinical data and pathological results of children with unilateral VTS who underwent surgical treatment at our center from July 2012 to July 2023. The children were categorized into the testicular excision group and testicular preservation group based on the surgical approach. Clinical characteristics and outcomes were compared between the two groups. Pathological examination results of excised testicular tissues were collected and analyzed, and long-term follow-up was conducted. A total of 368 children were included in this study. The age of the children at the time of surgery was 27 months (range, 6–156). Among them, 267 cases (72.6%) had VTS on the left side, and 101 cases (27.4%) on the right side. There were no statistically significant differences (P > 0.05) in age, affected side, contralateral testicular hypertrophy (CTH), testicular location, and preferred surgical incision between the testicular excision group (n = 336) and the testicular preservation group (n = 32). In the preservation group, two children experienced scrotal incision infections, showing a statistically significant difference compared to the excision group (P < 0.05). Pathological examination of excised tissues revealed fibrosis as the most common finding (79.5%), followed by vas deferens involvement (67%), epididymis involvement (40.5%), calcification (38.4%), and hemosiderin deposition (17.9%). Seminiferous tubules (SNT) was present in 24 cases (7.1%), germ cells (GC)in 15 cases (4.5%), and ectopic adrenal cortical tissue(EACT) in 1 case (0.3%). VTS belongs to a type of non-palpable testes (NPT) and requires surgical exploration. Considering the risk of scrotal incision infection after preserving atrophic testicular remnants and the unpredictable malignant potential, we recommend excision.

## Introduction

Cryptorchidism or undescended testis is one of the most common congenital anomalies in male newborns, with an incidence of 1.0–4.6% in full-term infants and up to 45% in premature infants^1^. For ease of surgical management, clinical practice often categorizes testes as palpable or non-palpable, with approximately 20% falling into the non-palpable category^2^. Vanishing testes syndrome (VTS) is one of the reasons of non-palpable testes (NPT) in children, characterized by the discovery of blind-ended spermatic vessels or nodules with unidentifiable normal testicular morphology during surgical exploration^1–3^. However, consensus on the optimal diagnostic and therapeutic approaches for VTS remains elusive. Some scholars previously advocated for surgical excision of testicular remnants, positing that residual testicular tissues may harbor germ cells (GC) and seminiferous tubules (SNT) with potential malignant transformation risks^4,5^. Nevertheless, recent research has presented an alternative perspective, suggesting that excision may not be imperative, given the relatively low incidence of GC and SNT, coupled with insufficient robust evidence indicating a continued presence of GC and SNT leading to potential malignant risks^3,6^. To delve further into this matter, the current study conducts a retrospective analysis of clinical and pathological data from 368 cases of unilateral VTS treated at our center over the past 11 years, aiming to provide additional data support for the diagnosis and treatment of VTS.

## Materials and methods

We conducted a retrospective analysis of case data for children with unilateral VTS who underwent surgical treatment at our center from July 2012 to July 2023, utilizing the electronic medical records system. Inclusion criteria comprised initial diagnosis of cryptorchidism, with surgical confirmation of blind-ended spermatic vessels or identification of atrophic nodules during exploration. Exclusion criteria encompassed a history of surgery on the affected side for undescended testis, testicular torsion, or incarceration related to inguinal hernia, all of which could lead to an NPT. A detailed dataset was constructed by carefully reviewing clinical records, surgical records, and pathological data for each patient.

### Preoperative ultrasound and definition of testicular hypertrophy

All patients underwent preoperative ultrasound examination of the scrotum or inguinal region to detect NPT. Simultaneously, the maximum longitudinal diameter of the contralateral testis was measured to assess whether it was hypertrophic. We utilized a criterion of 1.6 cm for testicular diameter in patients under 36 months as the threshold for defining testicular hypertrophy^3,7^.

### Surgical management

Prior to the commencement of surgical exploration, a thorough physical examination was conducted under general anesthesia to ensure that the patients met the inclusion criteria. If palpable cord-like tissue or atrophic remnants are detectable in the inguinal or scrotal region, inguinal or scrotal incision exploration is the preferred approach. If no structures are palpable, or if there is contralateral inguinal hernia present, laparoscopic exploration is the first choice. During laparoscopic exploration, if no intra-abdominal testis is found, and the spermatic cord enters the closed internal ring, exploration of the inguinal or scrotal region is performed. At the distal end of the spermatic cord, a small fibrotic remnant is identified, with no visible normal testicular tissue. If macroscopically abnormal intra-abdominal testicular remnants are discovered during laparoscopic examination, we advocate for their removal. During the procedure, communication with the parents was reiterated to inform them about the testicular development, and based on parental choice, patients were categorized into the testicular excision group or preservation group. All excised residual specimens underwent histological examination with hematoxylin and eosin (H&E) staining.

### Statistical analysis

Statistical analyses were performed using SPSS Statistics, version 25.0 (IBM Corp., Armonk, NY, United States). Continuous variables were presented as mean ± standard deviation (SD) or mean (min–max), and categorical variables were presented as n (%). Comparisons were made using chi-squared or Fisher’s exact tests for categorical variables. All tests were two-tailed. A significance level of *P* < 0.05 was considered statistically significant.

### Ethical approval

This study obtained approval from the Ethics Committee of Anhui Provincial Children’s Hospital (Approval No. 116123S21), ensuring the privacy and confidentiality of all data. Patient participation was voluntary, and informed consent was obtained from parents prior to their children’s involvement, documented through signed informed consent forms. All the procedures involving human participants followed in the study were in accordance with the ethical standards of the institutional and/or national research committee and with the 1964 Helsinki declaration and its later amendments or comparable ethical standards.

## Results

This study included a total of 368 pediatric patients with VTS. The mean age of the patients at the time of surgery was 27 months (range, 6–156). Among them, 267 cases (72.6%) had VTS on the left side, and 101 cases (27.4%) on the right side. Thirty-three cases were concurrently associated with contralateral undescended testis or inguinal hernia. Color Doppler ultrasound revealed atrophic nodules in 102 cases (27.7%), with 16 located in the inguinal region and 86 within the scrotum. In 263 cases (71.5%) of patients under 36 months, 172 cases (65.4%) exhibited contralateral testicular hypertrophy (CTH), with ultrasound longitudinal diameters exceeding 1.6 cm. A total of 206 cases (56%) opted for laparoscopic exploration, including 18 cases with contralateral inguinal hernia. The remaining 162 cases (44%) preferred inguinal or scrotal incision exploration.

Based on parental choices during surgery, 336 patients chose testicular excision, while the remaining 32 opted for testicular preservation. Comparative analysis of data between these two groups, including patient age, affected side, CTH, testicular position, and preferred surgical incision, showed no significant differences (*P* > 0.05) in these aspects (Table 1). However, it is noteworthy that in the preservation group, two cases, aged 22 months and 30 months respectively, experienced scrotal incision infections postoperatively, showing statistical significance (*P* < 0.05). All patients did not undergo contralateral testicular orchidopexy.

**Table 1:** Comparison of general data of two groups.

Factors	Excision group (n = 336)	Preservation group (n = 32)	*t *(*x*^2^) value	*P-*value
Age at surgery (m)	27.5 ± 9.6	28.4 ± 8.2	1.208	0.350
Affected side			0.255	0.614
Left	245 (72.92%)	22 (68.75%)		
Right	91 (27.08%)	10 (31.25%)		
CTH (Age < 36 m)			0.193	0.660
Yes	156 (46.43%)	16 (50.00%)		
No	84 (25.00%)	7 (21.88%)		
Testicular position			1.037	0.535^a^
Intra-abdominal	10 (2.98%)	0 (0.00%)		
Inguinal canal	236 (70.24%)	21 (65.63%)		
Scrotum	90 (26.78%)	11 (34.37%)		
Preferred surgical incision			2.387	0.303
Laparoscope	192 (57.14%)	14 (43.75%)		
Inguina	56 (16.67%)	6 (18.75%)		
Scrotum	88 (26.19%)	12 (37.50%)		
Incision infection				0.007^ab^
Yes	0 (0.00%)	2 (6.25%)		
No	336 (100%)	30 (93.75%)		

^a^Fisher’s exact test.

^b^*P* < 0.05.

**Figure 1:**
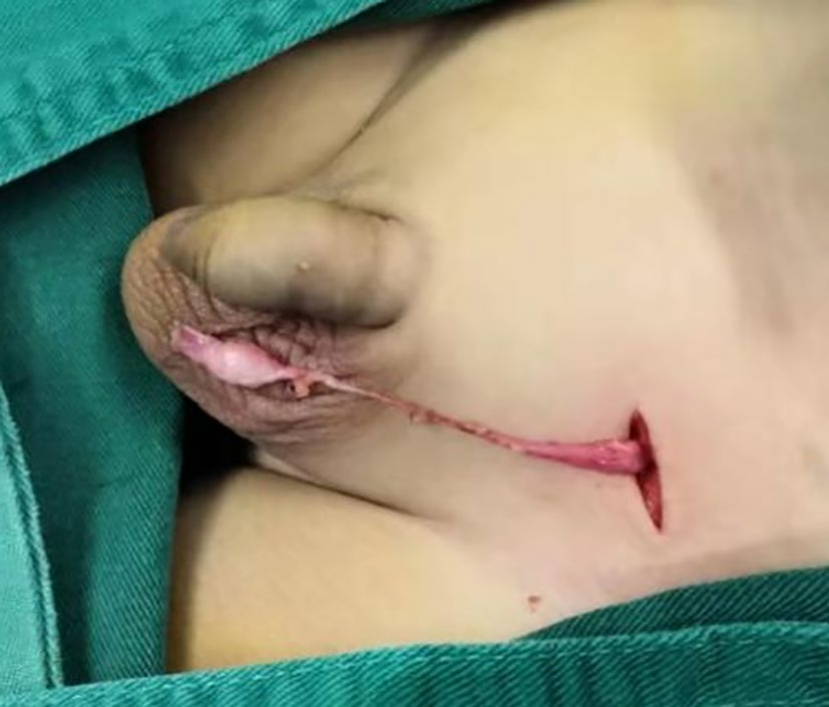
Atrophic nodule extracted via left inguinal incision.

**Figure 2:**
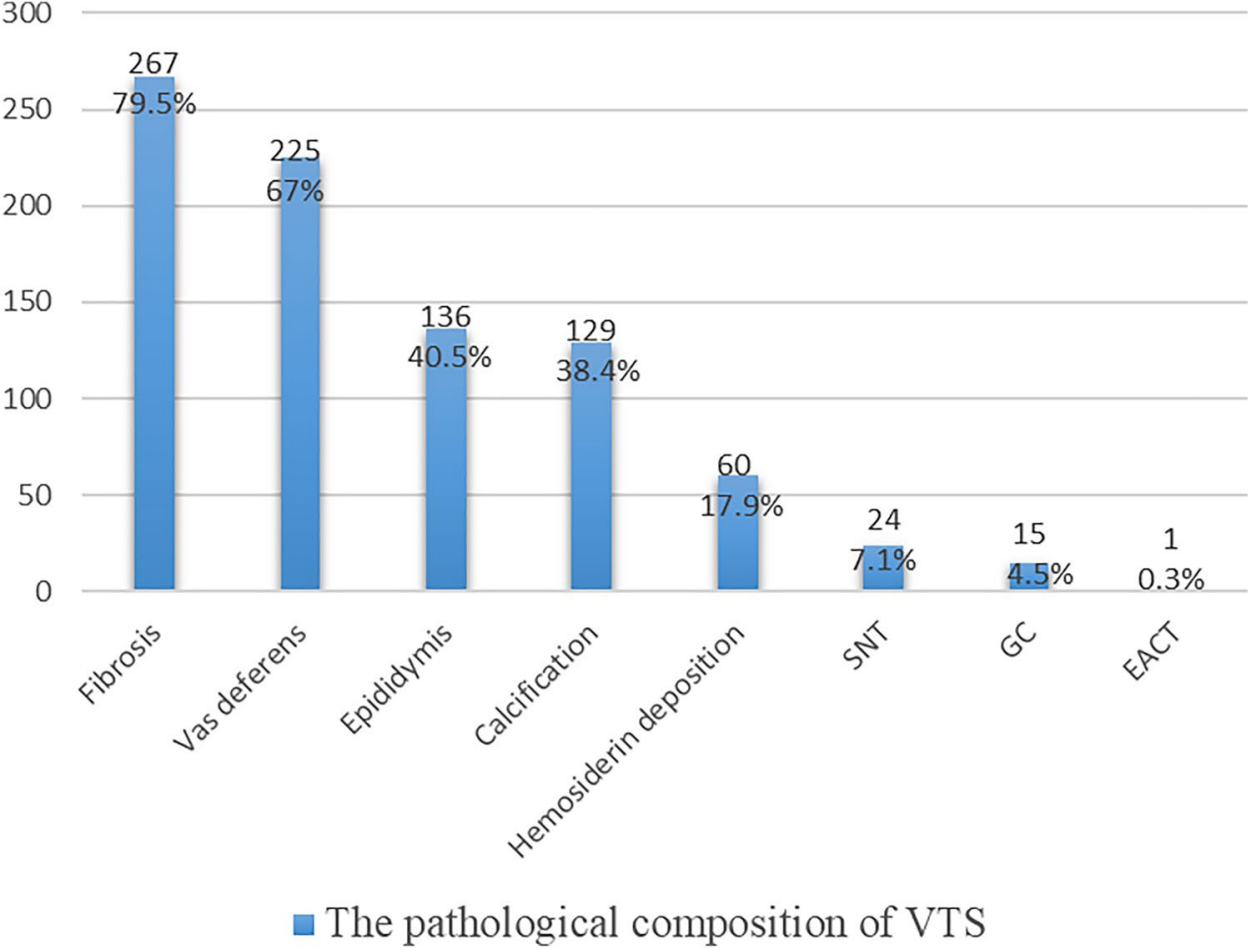
The pathological composition of VTS.

**Figure 3:**
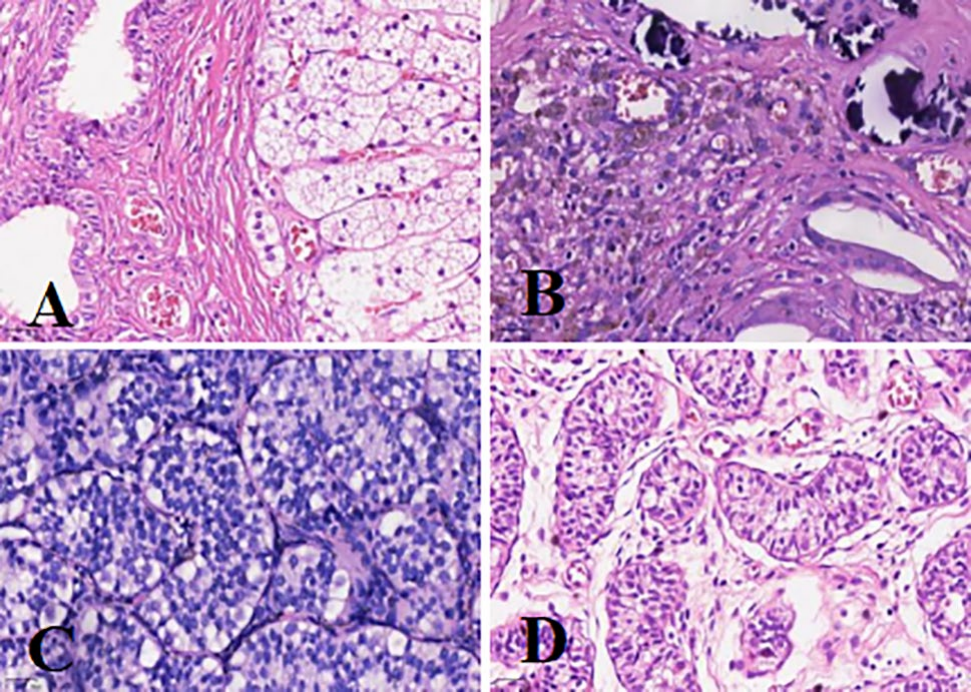
The pathological microscopic images of the excised tissue with H&E staining, captured under a × 200 magnification. (**A**) Ectopic adrenal cortex components are observed adjacent to the epididymal duct. (**B**) Iron-hemosiderin deposition and calcification are visible within the fibrous tissue. (**C**) Large and primitive germ cells are seen between the supporting cells of convoluted seminiferous tubules. (**D**) Underdeveloped, small convoluted seminiferous tubules.

**Figure 4:**
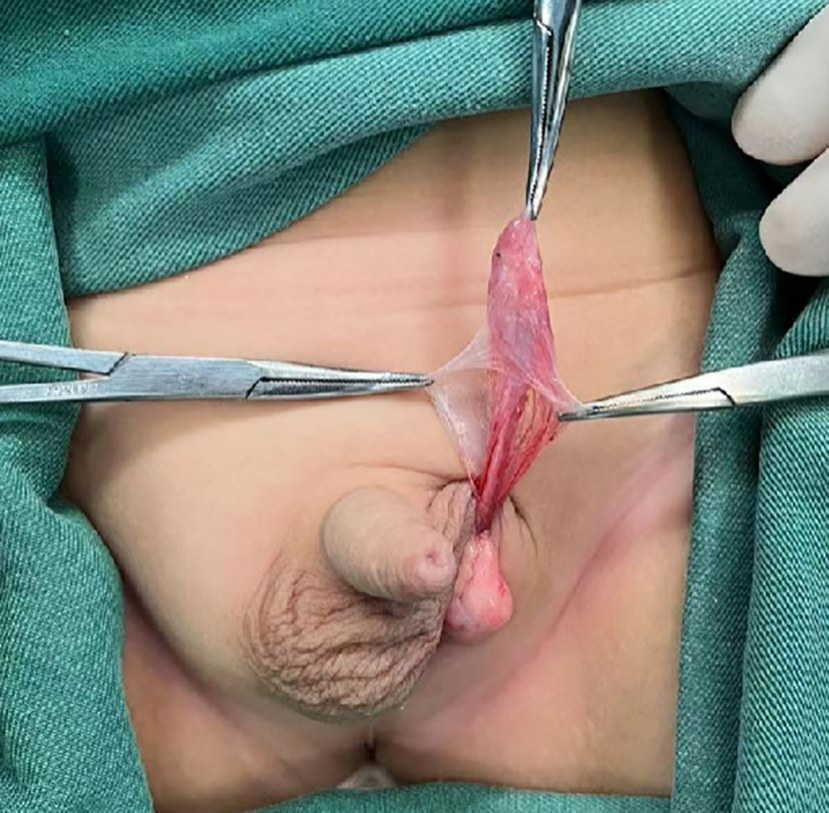
Atrophic nodule retrieved from the left scrotal incision.

Figure 1 illustrates an atrophic nodule retrieved from the left inguinal incision, where the spermatic vessels and vas deferens were absent, replaced by fibrotic strands (Fig. 1). Excision and pathological examination were performed. Results from H&E staining revealed fibrosis in 267 cases (79.5%), vas deferens involvement in 225 cases (67%), epididymis involvement in 136 cases (40.5%), calcification in 129 cases (38.4%), and hemosiderin deposition in 60 cases (17.9%) (Fig. 2). Multiple pathological components were found in 83.9% (282/336) of the excised tissue in the excision group. Additionally, 24 specimens (7.1%) showed the presence of SNT, while 15 specimens (4.5%) exhibited GC. Furthermore, one specimen revealed ectopic adrenal cortical tissue (EACT) (Fig. 3). An atrophic nodule retrieved from the left scrotal incision, showing the presence of spermatic vessels and vas deferens but lacking normal testicular parenchyma. After consulting with the parents, the remnant was preserved (Fig. 4).

Through outpatient follow-up examinations, with an average follow-up time of 40 months (range, 3–120), no abnormal development or testicular torsion was observed in the contralateral testis. Among the 32 patients in the preservation group, ultrasound examinations did not detect any malignant transformations, and no cases required secondary surgical excision.

## Discussion

Currently, the guidelines of the European Association of Urology categorize undescended testes into palpable and non-palpable types for clinical management. Among the 20% of NPT, 50–60% are intra-abdominal, canalicular, or peeping (right inside the internal inguinal ring), while the remaining 20% may be absent, 30% atrophied or underdeveloped, and sometimes include cases of ectopic testicles^1^. A retrospective review of clinical cases reveals that VTS is the fundamental cause in 35–69% of clinically NPT^8–10^. Therefore, some scholars consider that VTS is one of the reasons for the inability to palpate the testicle and recommend explaining this to all patients and their families before surgery^10^.

Due to the lack of consensus, various definitions of VTS exist in the literature^3–5,11,12^. The common feature is the presence of NPT, with only residual structures detected during surgical exploration. In this study, we adopt the definition of VTS as a congenital disease where normal testicular tissue is not identifiable by the naked eye after clinical exploration of the NPT^3,6,13^. This includes the presence of testicular remnants, nodules, or tissues adjacent to the testis/testicular side at the end of the spermatic cord. Although the etiology, pathological tissue composition, and prognosis of VTS may differ from those of surviving undescended testes^5,6^, VTS is considered a result of prenatal or late gestational vascular thrombosis, torsion, or endocrine abnormalities^5^. However, recent studies strongly support the theories of vascular accidents and prenatal torsion, rather than endocrine diseases^14^. Our study consistently observes and supports this theory, as fibrosis, malnutrition-induced calcification, hemosiderin deposition, and the presence of macrophages in the excised residual tissues indicate vascular accidents^4,15,16^. Additionally, VTS is more common on the left side, as reported in previous studies^3–6,16^. He et al.^16^ suggest that it is challenging to explain this significant lateralization with endocrine factors. The potential reasons include anatomical relationships between the left testicular vein and the left renal vein, leading to vascular occlusion secondary to abnormal renal activity^5^. Furthermore, the earlier descent of the left testis during fetal development may be another contributing factor^15,16^.

Preoperative imaging examinations are unable to determine the presence of the testes^17^. Although ultrasound is a non-invasive tool, it is time-consuming, expensive, and lacks accuracy in detecting the presence or location of intra-abdominal testes in non-palpable cases^1,18^. However, Press et al.^19^ found that ultrasound can detect 84.8% of NPT, including 12.1% with atrophic nodules. These results can impact surgical planning, avoiding unnecessary laparoscopic explorations. In our study, ultrasound is cost-effective (approximately $13), and all patients underwent preoperative ultrasound examinations. The ultrasound results revealed 102 cases (27.7%) with atrophic nodules, including 16 cases in the inguinal region and 86 cases in the scrotum. When ultrasound cannot detect the testes, parents are informed of various possibilities and corresponding treatment methods for the presence of NPT. Another essential role of ultrasound is detecting CTH. Unlike measuring tools such as calipers, ultrasound can exclude interference from the epididymis, scrotal skin, and adjacent soft tissues during the measurement, making it considered the best tool for assessing testicular size in the pediatric population^20^. Although CTH often indicates ipsilateral testicular atrophy, ultrasound is often challenging in determining the location of atrophic nodules^16^. Therefore, CTH has no impact on the decision-making for NPT management, consistent with the results of Wei et al.^21^. Another study’s findings indicate that regardless of ultrasound results, over 80% of surgeons choose laparoscopic exploration as the initial step in diagnosing and treating NPT, while less than 20% of participants opt for inguinal/scrotal approaches as the primary choice for NPT. Supporters of this approach believe that, as only 14–32% of patients have an intra-abdominal testis, this incision allows for easier detection of the testis, potentially avoiding the need for laparoscopic exploration^22^. However, if there is suspicion of a nodular lesion on the same side and CTH is present on the contralateral side, the option of scrotal incision and excision of the nodule can be considered to confirm testicular disappearance, thus obviating the need for laparoscopic exploration^1^. In our study, 206 cases (56%) of patients who did not initially exhibit cord-like structures or atrophic remnants in the inguinal or scrotal region underwent initial laparoscopic exploration. Additionally, 162 cases (44%) with detected nodules in the inguinal or scrotal region underwent direct inguinal or scrotal exploration and residual excision. This surgical approach is considered to have potential certainty for the diagnosis and treatment of testicular disappearance^23^.

Past studies have debated whether atrophic testicular nodules need to be excised. Unlike undescended testes, congenital undescended testes increase the risk of future malignancies^24^. However, the etiology of VTS differs from congenital undescended testes, and therefore, the risk of future malignant tumors may also differ^6^. So far, there has been only one isolated report of intratubular germ cell neoplasia (ITGCN) in residual testicular ducts in a 9-year-old child, but it lacked immunohistochemical support^25^. The controversy over whether to excise atrophic nodules arises from different reported rates of GC occurrence^3–5,10,16^ and the subsequent risk of malignancy.A large-scale retrospective study conducted until now indicated an overall incidence rate of 3.1% for GC and 6.6% for SNT^16^. However, a recent systematic review revealed that the overall incidence rate of GC is 5.3%, and SNT is 10.7%^6^. In our study, considering the potential medical disputes associated with excising testicular tissues and the unpredictable malignant potential of retaining atrophic testicular nodules, parents were allowed to observe the atrophy of the testicles during surgery and choose between excision and fixation. Based on this, we divided the patients into excision and fixation groups. There were no significant differences between the two groups in terms of patient age, affected side, CTH, testicular location, and preferred surgical incision. However, the fixation group had two cases of postoperative scrotal incision infections, which showed statistical significance. In the excision group, we found an SNT occurrence rate of 7.1% and a GC occurrence rate of 4.5%. Postoperative follow-up did not reveal abnormal development or torsion of the contralateral testicle. In the fixation group, no malignant conditions were detected by ultrasound, and there were no cases requiring a second excision surgery. At the same time, we observed that 336 cases (91.3%) of patients underwent excision surgery, a relatively high proportion that may reflect a preference for surgical interventions within parents. Despite our efforts to provide objective information regarding treatment options, we cannot rule out the potential influence of personal preferences within the medical team and concerns from parents about future risks on the decision-making process. Interestingly, in this study, we discovered a case of EACT. First discovered by Morgagni in 1740^26^, EACT can be found anywhere along the migration path from the urogenital ridge during embryonic development. While descriptions of EACT have been reported in the abdomen, broad ligament, testicles, spermatic cord, kidneys, and retroperitoneum^11^, it is rarely reported in atrophic testicular nodules. Although the number of reported cases is limited, EACT is generally accepted to have malignant potential^11,27^, thus recommending excision.

By reviewing the literature, the low occurrence rates of SNT and GC and insufficient evidence supporting the continuous existence of future malignant potential lean towards the argument that surgical excision is unnecessary^3,6,10^. However, this overlooks the clinical significance of surgical procedures. VTS is a type of NPT, often requiring surgical exploration to determine the presence of viable testes. Considering the risk of scrotal incision infections after retaining atrophic nodules and the unpredictable malignant potential, even if the likelihood of malignancy is extremely low, parents find it difficult to accept. Therefore, we recommend excision.

Despite providing valuable data, this study has some limitations. Firstly, it has the limitations inherent in retrospective research, such as information completeness and selection bias. Secondly, the identification of SNT and GC in this study relied on H&E staining and lacked immunohistochemical results. Additionally, the follow-up period in this study was relatively short, requiring long-term follow-up of the malignant potential after retaining atrophic testicular nodules. Therefore, future prospective randomized controlled studies with long-term follow-ups will be more valuable.

In conclusion, in this study, SNT was found in 24 cases (7.1%) of excised atrophic remnants, GC in 15 cases (4.5%), and EACT in 1 case (0.3%). Although there is not sufficient robust evidence to prove the sustained malignant potential of GC and SNT over time, considering that VTS is a type of NPT requiring surgical exploration, and taking into account the risk of incision infection and the unpredictable malignant potential after retaining atrophic testicular remnants, which, even if low, is difficult for parents to accept, we recommend excision.

## Data Availability

The datasets used and/or analysed during the current study available from the corresponding author on reasonable request.

## Abbreviations

VTS: Vanishing testes syndrome
CTH: Contralateral testicular hypertrophy
SNT: Seminiferous tubules
GC: Germ cells
EACT: Ectopic adrenal cortical tissue
NPT: Non-palpable testes
H&E: Hematoxylin and eosin
SD: Standard deviation
ITGCN: Intratubular germ cell neoplasia
